# Oxidized (non)-regenerated cellulose affects fundamental cellular processes of wound healing

**DOI:** 10.1038/srep32238

**Published:** 2016-08-25

**Authors:** M. U. Wagenhäuser, J. Mulorz, W. Ibing, F. Simon, J. M. Spin, H. Schelzig, A. Oberhuber

**Affiliations:** 1 Department of Vascular and Endovascular Surgery, University Hospital Düsseldorf, Moorenstraße 5, 40225 Düsseldorf, Germany; 2 Division of Cardiovascular Medicine, Stanford University School of Medicine, Stanford University, Stanford, CA 94305, USA

## Abstract

In this study we investigated how hemostats such as oxidized regenerated cellulose (ORC, TABOTAMP) and oxidized non-regenerated cellulose (ONRC, RESORBA CELL) influence local cellular behavior and contraction of the extracellular matrix (ECM). Human stromal fibroblasts were inoculated *in vitro* with ORC and ONRC. Cell proliferation was assayed over time, and migration was evaluated by Live Cell imaging microscopy. Fibroblasts grown in collagen-gels were treated with ORC or ONRC, and ECM contraction was measured utilizing a contraction assay. An absolute pH decline was observed with both ORC and ONRC after 1 hour. Mean daily cell proliferation, migration and matrix contraction were more strongly inhibited by ONRC when compared with ORC (p < 0.05). When control media was pH-lowered to match the lower pH values typically seen with ORC and ONRC, significant differences in cell proliferation and migration were still observed between ONRC and ORC (p < 0.05). However, in these pH conditions, inhibition of matrix contraction was only significant for ONRC (p < 0.05). We find that ORC and ONRC inhibit fibroblast proliferation, migration and matrix contraction, and stronger inhibition of these essential cellular processes of wound healing were observed for ONRC when compared with ORC. These results will require further validation in future *in vivo* experiments to clarify the clinical implications for hemostat use in post-surgical wound healing.

Vascular surgery patients are at elevated risk to develop superficial and deep incisional surgical site infections, with increased morbidity leading to prolonged hospital stays. These complications are common, with an overall risk of 3–44%, leading to enormous costs for health systems^1^,^2^,^3^. Thus accelerated, or at least normalized, surgical wound healing in these patients would be greatly beneficial^4^,^5^.

Wound healing is hierarchically regulated based on an interplay between cellular, humoral and molecular mechanisms, and begins immediately after wounding^6^. The process is often subdivided into 3 independent phases, which partially overlap^7^. Initially there is an inflammatory phase, during which pro-inflammatory cytokines are released, leading to the recruitment of pro-inflammatory cells^8^,^9^. In this milieu, keratinocytes and fibroblast begin to proliferate within 3–4 days, establishing a collagen-rich granulation tissue^10^. After the proliferation phase, remodeling slowly transforms the granulation tissue into robust scar tissue. This final process can continue for years^8^,^10^.

Chemical hemostats are often used by surgeons for local bleeding control after performing vascular anastomoses. Although, newer materials such as plasma-derived thrombin and fibrinogen have shown satisfying results, cellulose-based hemostats are still commonly used, as they are highly cost-effective^11^. Cellulose is a homopolysaccharide which is created through polymerization of glucopyranose through β-glucosidic bonds^12^. The manufacturing process differs slightly between oxidized regenerated cellulose (ORC), in which organized fibers are formed prior to oxidation, and oxidized non-regenerated cellulose (ONRC). Oxidation of cellulose is typically performed using dinitrogen tetroxide^13^. The primary component of oxidized cellulose is polyuronic acid, which is rapidly processed by glycosidases using β-elimination *in vivo*; however non-oxidized hydroxyl groups remain as a fibrous component which require phagocytosis by macrophages prior to hydrolysis^14^. However, the effect of *in situ* remaining hemostats on the postoperative wound healing process is poorly investigated so far.

The objective of this study is to compare an ONRC-based hemostat (RESORBA CELL, Resorba, Nuremberg, Germany) with an ORC-based hemostat (TABOTAMP, Norderstedt, Germany) regarding their effects on fundamental processes of deep incisional wound healing (DIWH), including cell proliferation and migration, and contraction of the extracellular matrix (ECM).

## Results

Postoperative wound healing is highly dependent on the interaction between different cell lines; however, fibroblasts seem to be of particular relevance for DIWH. For that reason, human fibroblasts were used for analyzing key cellular sub-processes. As degradation of ORC and ONRC initially consists of hydrolysis with release of acidic groups, we investigated the pH-value time course for both hemostats.

**Figure 1:**
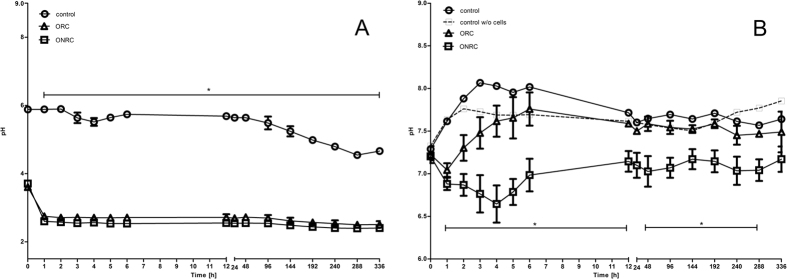
pH-value time course. (**A)** Absolute pH value of normal saline (NaCl 0,9%) during ORC and ONRC exposition. Decline in pH value occurred during the first hour before equilibration. No difference in total pH value decline between ORC and ONRC. (**B)** pH values of cell culture medium (without w/o cells) in a human fibroblast cell culture. Both hemostats had lower pH values compared to controls (*p < 0.05 between 1–5 h and at 240 h). ONRC showed lower pH value throughout the investigation period compared to ORC (*p < 0.05; one-way ANOVA with Bonferroni post test). n = 12.

A significant decline in absolute pH was observed for ONRC and ORC compared to controls. Over the first hour there was a significant pH drop with both hemostats in normal saline. After 1-hour absolute pH value remained constantly at approximately 2.5 for ONRC and ORC (Fig. 1A) (p < 0.05). The results showed a significant lower pH value for ONRC throughout the whole investigation period (p < 0.05). We observed opposite tendencies in pH value trend between 1–4 hours, as ONRC continued to lower the local pH, while ORC followed the pH value trend of controls and exhibited a gradual increase (Fig. 1B).

**Figure 2:**
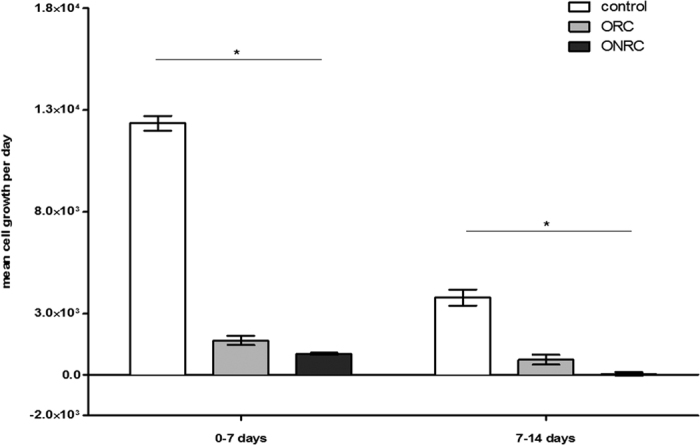
Mean cell growth rate. Mean cell growth rate was determined using WST-1 assay. Total cell number was measured at day 7 and 14. Mean cell growth rate per day was calculated for the first and second week separately. Significant inhibition of cell proliferation for ORC and ONRC, stronger inhibition for ONRC compared to ORC for both investigation periods. (*p < 0.05; Kruskal-Wallis test with Dunn’s post test). n = 12.

We also found that mean daily proliferation of fibroblasts was lower during the first week for ONRC (1.0 × 10^3^ Δcells/day ± 180 Δcells/day) and ORC (1.6 × 10^3^ Δcells/day ± 790 Δcells/day) as well as for the second week (ONRC: 32 ± 320 Δcells/day; ORC: 730 ± 840 Δcells/day) when compared to controls (1.2 × 10^4^ ± 1.3 × 10^3^ Δcells/day: 3.8 × 10^3^ ± 1.4 × 10^3^ Δcells/day) (p < 0.05). Overall, ONRC displayed lower mean daily cell proliferation compared to ORC over both weeks (p < 0.05). There was near-total inhibition of cell proliferation within the second week for ONRC, whereas proliferation remained similar for ORC over the full duration (Fig. 2).

**Figure 3:**
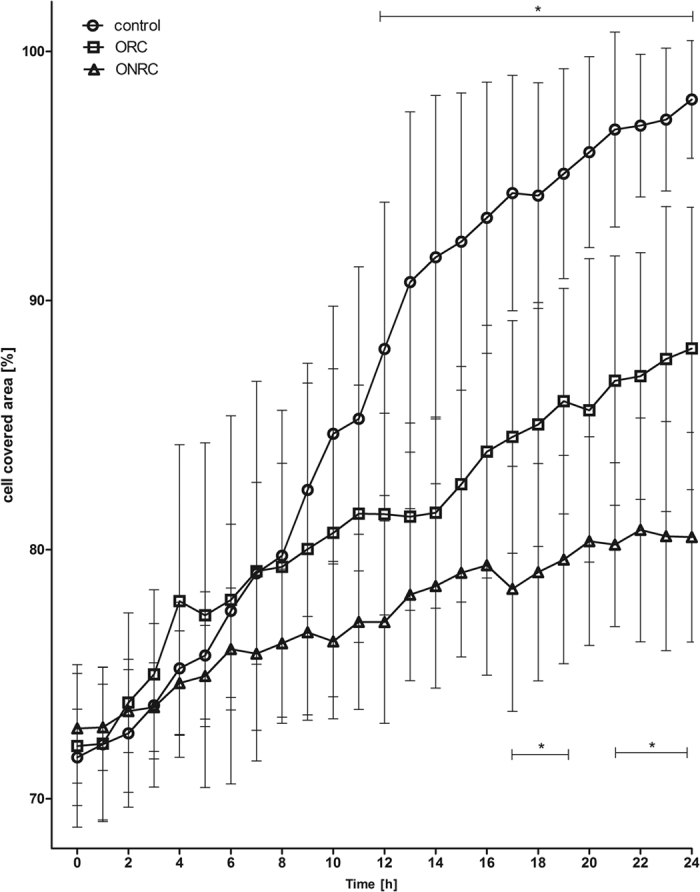
Cell migration. Shown is the cell covered area between two cell fractions using a cell migration assay. Inhibition of cell migration for ORC and ONRC. ONRC showed slower cell migration compared to ORC (*p < 0.05; one-way ANOVA with Bonferroni post test; significant difference between all study groups; — significant difference between both hemostats and controls). n = 12.

Cell migration is essential for functional DIWH. We found that both hemostats inhibited cell migration compared to controls. Throughout the investigation period, cell migration was more strongly inhibited by ONRC compared to ORC. Differences between the hemostats were statistically significant for the periods between 17–19 hours and between 21–24 hours (p < 0.05). After 24 h, coverage of the inter-chamber gap was 98.1% ± 2.3% for controls, and 88.1% ± 5.7% and 80.5% ± 4.2% for ORC and ONRC respectively (Fig. 3).

**Figure 4:**
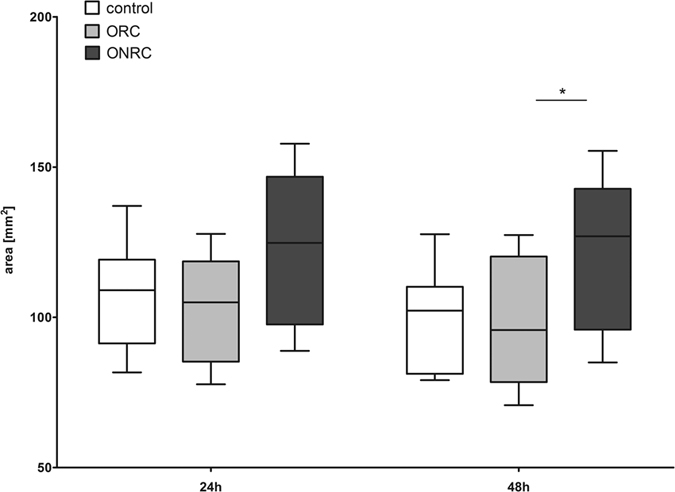
Contraction of the extracellular matrix. Shown is the surface area of 1%-collagen matrices after 24 and 48 hours. While ORC showed no differences compared to controls at both time points, ONRC demonstrated weaker matrix contraction compared to ORC (*p < 0.05; Kruskal-Wallis test with Dunn’s post test). n = 12.

Contraction of the ECM is also indispensable for development of mature scar tissue. Our contraction assay results illustrate that nearly all observed contraction in matrix surface area occurred within the first 24 hours. Surface area of ORC matrices decreased ~25% ± 8.9%, within 24 hours, and ~16% ± 10.1% for ONRC. Thus, ONRC (24 h: 123 mm^2^ ± 24.3 mm^2^; 48 h: 127 mm^2^ ± 24 mm^2^) matrices showed a greater surface area after 24 and 48 hours compared to ORC (24 h: 103.9 mm^2^ ± 17.9 mm^2^; 48 h: 98.6 mm^2^ ± 20.2 mm^2^) and controls (24 h: 107.4 mm^2^ ± 18 mm^2^; 48 h: 99.7 mm^2^ ± 17 mm^2^) (p < 0.05). Differences between ORC and controls were not significant at either time point (Fig. 4).

**Figure 5:**
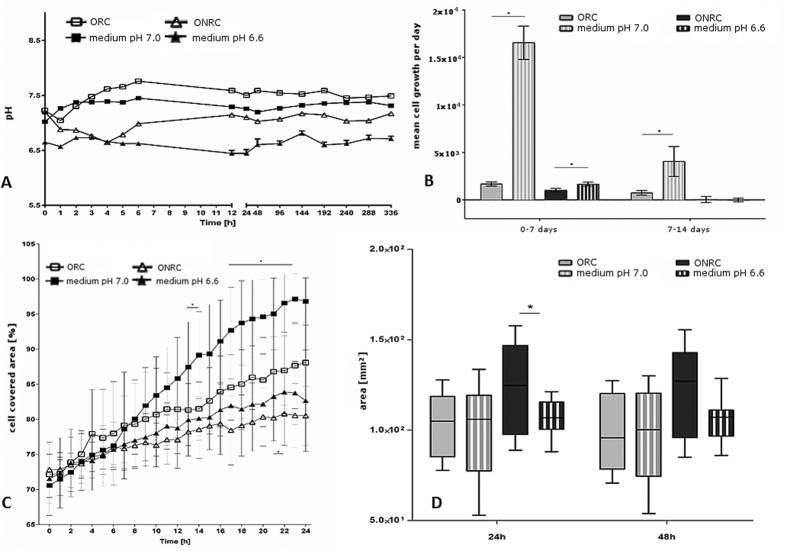
pH value, mean cell growth, cell migration and matrix contraction compared to pH-lowered cell media. (**A)** pH value of fibroblast cultures with pH-lowered control media compared to ORC and ONRC. A difference in pH of 10% for each measuring point was accepted for experiments. (**B)** Shown is the mean cell growth rate for the first and second week. Inhibition of cell proliferation for ORC and ONRC compared to pH-lowered controls in the first week, for ORC for the second week (*p < 0.05; Mann-Whitney U test). n = 12. (**C)** Cell migration for ORC and ONRC compared to pH-lowered controls as percentage of a cell covered area between two cell fractions. Inhibition of cell migration for ORC and ONRC compared to controls (*p < 0.05; multiple t-test with Bonferroni correction). n = 12. (**D)** Contraction of the extracellular matrix for ORC and ONRC compared to pH-lowered controls. ORC showed almost no difference at both measuring points compared to pH-lowered controls, whereas ONRC outlined inhibited matrix contraction. (*p < 0.05; Mann-Whitney U test). n = 12.

To investigate hemostat-based effects that might be due solely to acidosis/pH changes, we used low pH-media, imitating the values achieved during the hemostat time-course (Fig. 5A). The low-pH control media was set initially at pH 7.0 for ORC (“medium pH 7.0”) and at pH 6.6 for ONRC (“medium pH 6.6”) as per the initial pH value measurements (Fig. 1B). Again cell proliferation was decreased for both ONRC and ORC within the first week compared to low-pH-media controls (medium pH 6.6: 1.6 × 10^3^ Δcells/day ± 220 Δcells/day; medium pH 7.0: 1.6 × 10^4^ Δcells/day ± 1.7 × 10^3^ Δcells/day) (p < .05). During the second week, this effect could only be seen for ORC (ORC: 730 ± 840 Δcells/day; medium pH 7.0: 4.0 × 10^3^ Δcells/day ± 1.6 × 10^3^ Δcells/day) (p < 0.05) (Fig. 5B). Cell migration studies revealed that ORNC and ORC displayed enhanced inhibition of migration compared to low-pH-media controls, and the difference was accentuated after 6 hours in both study groups. Results were statistically significant between 13–14 and 17–23 hours for ORC and between 21–22 hours for ONRC (p < 0.05). After 24 h, controls with medium pH 7.0 showed 96.8% ± 3.4% cell coverage of the inter-chamber space, while controls with medium pH 6.6 showed 82.7 ± 7.2% coverage (Fig. 5C). Matrix contraction studies identified no differences for ORC compared to low-pH controls at 24 hours (ORC: 103.9 mm^2^ ± 17.9 mm^2^; medium pH 7.0: 104.6 mm^2^ ± 24.9 mm^2^) or 48 hours (ORC: 97.6 mm^2^ ± 20.2 mm^2^; medium pH 7.0: 103.2 mm^2^ ± 24.7 mm^2^). In contrast, there was significant inhibition of contraction for ONRC compared to low-pH controls at 24 hours (ONRC: 125 mm^2^ ± 24.3 mm^2^; medium pH 6.6: 107.1 mm^2^ ± 9.3 mm^2^) (p < 0.05), and near-significance at 48 hours (ONRC: 128.5 mm^2^ ± 24 mm^2^; medium pH 6.6: 107.6 mm^2^ ± 11.4 mm^2^) (p = 0.052) (Fig. 5D).

## Discussion

Cellulose-based hemostats are known for high clinical convenience, as they are storable at room temperature and can be quickly deployed or removed. The macroscopic appearance of RESORBA CELL is more frayed at the edges, and overall more flexible compared to TABOTAMP. These agents are primarily used for small amounts of bleeding^15^,^16^,^17^. Lewis *et al*. describe superior hemostasis for ONRC, with equivalent bactericidal effectiveness when compared to ORC^13^. Both materials have already proven beneficial antibacterial effects even against antibiotic resistant microorganisms such as VRE, MRSA^13^,^18^. Little is known regarding how ORC and ONRC influence DIWH. Therefore, we investigated the effects of ONRC and ORC on fibroblast cell proliferation, migration, and ECM contraction after hemostat degradation *in vitro* to clarify their potential impact in impaired DIWH.

As degradation of both hemostats results in a release of acidic groups, we investigated the effect of ORC and ONRC on the pH value over time. In the absence of cells and cell culture media, there was a sudden drop in pH value within the first hour for both hemostats. After this initial decline, no further changes in pH were noted; however, we later observed a marginally lower pH for ONRC. Next, we modeled the pH values observed in humans *in vivo* in the presence of fibroblasts utilizing cell cultures. ONRC showed lower pH values in cell culture throughout the investigation period. The hemostats showed opposite effects on pH value, particularly in the time period between 1–4 hours. Within this time frame, ONRC continued to lower the pH value whereas ORC showed an increase in pH value, similar to non-hemostat controls. Two different hypotheses might explain this behavior. The observed effect could be based on the greater surface area of the frayed fibers found in ONRC. This might lead to an increased release of acidic-groups per time unit, exceeding the buffering capacity of the culture media and lowering the pH value^13^. We suspect the differences seen between fibroblast cell cultures and normal saline experiments may be due in part to the logarithmic nature of the pH scale. Chemical buffers in culture media tend to hold the pH value more towards the middle of the scale, where changes in [H+] would have less impact on the pH value than at the end of the scale. Evaluation of this theory will require that material input and surface area be tightly controlled in future experiments. One possible approach to this would be to normalize the hemostat input to its density and pestle the hemostats thereafter. In this way, the release of acidic groups and changes in pH value could be investigated independently from the different working surfaces of both hemostats.

Another possible explanation of the divergence in pH value in cell culture media of fibroblast cultures between the hemostats might be that alterations occur in the cellular function of fibroblasts, meaning the fibroblasts themselves might acidify the environment. *In vivo* degradation of cellulose-based hemostats was studied by Pierce *et al*. and Dimitrijevich *et al*., who showed that beyond enzymatic degradation there is a fibrous component of hemostats which requires phagocytosis by macrophages for complete clearance^14^,^19^. We believe that these fibrous components might alter cell function, leading fibroblasts to release acid or more likely to apoptose. The vicious cycle of acidosis causing increased apoptosis leading to further acidic group release has been described previously, and might explain our findings^20^. Future experiments would benefit from investigations of the effects of these hemostats and the associated pH phenomena on fibroblast apoptosis. As we only observed the pH effect for ONRC, we suspect it might be ONRC specific.

In this study we also modulated the pH value to investigate whether hemostats themselves, rather than the associated changes in pH value could be responsible for the effects of both hemostats on cell proliferation, migration and matrix contraction. To investigate this, we elected to lower the pH value of the controls rather than adding buffer to the hemostat samples, as total depletion of buffer capacity due to massive acidic group release over a short time might also occur *in vivo*. For that reason, this approach might better mimic *in vivo* conditions. More importantly, depletion of buffer capacity might even be essential to the findings we observed. For example, fibrous degradation components might have variable influences at different pH values.

Fibroblast function is crucial for several aspects of healthy post-surgical wound healing. Overcoming the spatial defects through generation of cell-rich tissue is aided by adequate cell proliferation. We therefore studied the influence of ONRC and ORC on cell proliferation, and demonstrated that both hemostats inhibited fibroblast proliferation compared to controls. This effect was larger for ONRC compared to ORC. Froehlich *et al*. described reduced fibroblast proliferation at lower pH values, as there is a greater tendency towards contact-inhibition and decreased sensitivity to growth stimulation once confluent^21^. Our low-pH culture media experiments suggest that effects of both hemostats on fibroblast proliferation are independent of their impact on pH alone. Akyol *et al*. and Liu *et al*. investigated the effect of ORC on fibroblast proliferation in animal models. Both authors described either no significant effect or even enhanced remodeling of the extracellular matrix, which they conclude resulted from increased cell proliferation^22^,^23^. It should be emphasized that these authors focused on either epithelial or mucosal wound healing reactions, which are somewhat different processes from this study. Epithelium and mucosa have high regenerative capacity, with a high basal proliferation rate, and multiple cell-cell interactions. This structure is histologically different from the way fibroblasts are organized in connective tissue, which seems to be the most relevant tissue in DIWH. Fibroblasts in different tissues might also react differently to external stimuli, as Varkey *et al*. demonstrated for fibroblast sub-populations within the skin^24^. Our studies focus on stromal fibroblasts, which appear to play a central role in DIWH. Clearly, the use of fibroblasts alone is not a perfect model to mimic *in vivo* conditions, where wound healing depends on multicellular interactions. Nevertheless, our results regarding hemostats and fibroblast proliferation stand in clear contrast to some published findings.

Fibroblast migration is also crucial for DIWH, helping to develop bands of connective tissue and establish immature granulation tissue. The current literature provides variable results with regard to the influence of hemostats on cell migration. Hart *et al*. found that wounds in diabetic animals treated with ORC/collagen sponges demonstrated significantly accelerated wound closure when compared to control wounds in similar animals. Histological analysis of the wound tissues suggested that using ORC/collagen sponges enhanced the formation and maturation of granulation tissue as a result of increased cell migration^25^. Rassu *et al*. showed that using ORC in oncoplastic breast surgery could promote dermal fibroblast proliferation and cell migration which resulted in beneficial effects for adjustment of the shape^26^. In contrast, Krishnan *et al*. described ORC as detrimental to wound healing *in vivo,* and uncovered no advantageous effects for ORC on cell proliferation and migration^27^. Notably, these *in vivo* results have limited applicability to our study. Their authors found that the prevention of postoperative hematoma led to increased cell proliferation, a process we did not investigate. Rather we concentrated on the direct inter-dependency between hemostats and cellular function. Moreover, the above authors used diabetic mice to study wound healing with cellulose hemostats. In that model, the antibacterial effect of hemostats might have outweighed the potential inhibiting effects on wound healing. Our study found that cell migration was inhibited *in vitro* for ORC and ONRC compared to controls. This effect remains after buffering controls to similar pH values produced by hemostats. Therefore, we suggest that there exists a material-based negative effect on fibroblast cell migration, which is more evident for ONRC compared to ORC.

A balanced level of wound contraction is essential for optimal healing. Fibroblasts promote wound contraction, through activation of signaling pathways promoting a shift from fibroblast to myofibroblast^28^. We found that ONRC inhibit matrix contraction, whereas we could not observe a difference between ORC and controls. These results suggest that ONRC in particular might be responsible for inhibition of matrix contraction, and again the findings appear independent of acidosis resulting from hydrolysis. They might be dependent on fibrous compounds, which we believe may differ significantly between ORC and ONRC, leading to prolongation of clearance. Such compounds may also differ in their resistance towards contraction forces, resulting in inhibited matrix contraction. It appears likely that ONRC-specific fibrous hydrolysis end-products suppress matrix contraction more than ORC derived end-products.

In conclusion, both ORC and ONRC inhibit fibroblast proliferation, and migration. These effects do not exclusively depend on acidosis, suggesting that material-specific compounds such as fibrous hydrolysis end-products are responsible. Further, ONRC inhibits matrix contraction, unlike ORC. ONRC may more strongly inhibit essential cellular sub-processes, which are fundamentally important for functional wound healing. However, these findings will require further validation using *in vivo* models before their clinical impact can be assessed.

## Methods

### Hemostats

Employed hemostats included RESORBA CELL (oxidized non-regenerated cellulose -ONRC) and TABOTAMP (oxidized regenerated cellulose - ORC).

### Cell culture

Human stromal fibroblasts were purchased from PromoCell (Heidelberg, Germany) and propagated using Dulbecco’s Modified Eagle Medium (Biochrom, Berlin, Germany) supplemented with 20% fetal bovine calf serum (FBS-Superior, Biochrom Berlin, Germany), and 10 Units/ml Penicillin/0,1 mg/ml Streptomycin (PAN Biotech). Conditions included 37 °C, CO_2_ 5%, with medium changes every second to third day. Medium changes were suspended during subsequent experiments – detailed below. Cells were subcultured at 80–90% confluence, using 0.05% trypsin/0.02% ethylenediaminetetraacetic acid (EDTA) (PAN Biotech, Aidenbach, Germany). Morphological cell assessment was performed using phase-contrast microscopy (Olympus CKX41, Olympus, Germany).

### pH value measurement

ONRC (RESORBA CELL) and ORC (TABOTAMP) hemostats were sliced into 1 × 1 cm sections using a sterile scissor (Aesculap AG, Tuttlingen, Germany). Sections were placed in normal saline (NaCl 0.9%) to study whether there was a difference in the acidifying behavior of the hemostats. To better imitate *in vivo* conditions, fibroblasts were seeded into 12-well plates (Greiner Bio-One, Solingen, Germany) and sections were placed in culture medium (total volume: 3 ml). The pH-value was measured at baseline and hourly for the first 1–6 hours, with further measurements being performed at 12 h, 48 h, 96 h, 144 h, 192 h, 240 h, 288 h and 336 h using a pH meter (FiveEasy^TM^Profi-Kit 20 ATC, MettlerToledo, Gießen, Germany).

### Cell proliferation

As described above, hemostat sections were placed in the medium of fibroblasts (4 × 10^3^) seeded in 12-well plates (Greiner Bio-One, Solingen, Germany). After 7 and 14 days, total cell number was measured using the WST-1 proliferation assay (Roche Life Scientific; Penzberg, Germany) according to the manufacturer’s instructions. Briefly, all culture media was removed and replaced by 450 μl of fresh medium, supplemented with 50 μl of WST-1 reagent. After incubation for 2 h, the supernatant was mixed at 500 rpm for 3 min using an orbital shaker (IK-Schüttler MTS4, IKA-Werke GmbH, Staufen, Germany). An aliquot of 100 μl of the supernatant was measured at 450 nm by microplate reader (Victor X4, PelkinElmer, Massachusetts, USA). Mean daily proliferation was calculated using the difference in total cell number between measuring points divided by days of cultivation.

### Matrix contraction

Fibroblasts at 10 × 10^6^ cells/ml were seeded in a collagen I matrix (5 mg/ml rat tail collagen 1, ibidi GmbH, Planegg, Germany). The concentration of collagen I in the matrix was set to 1.5 mg/ml. During gelation, the mixture was pipetted into 24-well plates (Greiner Bio-One, Solingen, Germany) and supplemented with culture medium to a final volume of 300 μl. Mixtures were then placed in a cell culture incubator (HERAcell240, Heraeus, Hanau, Germany) for 30 minutes. 1 × 1 cm hemostat slices were placed in the supernatant and an additional 2 ml of culture medium were added.

After 24 h, the adherent matrices were gently detached using a sterile spatula and photographed (ChemiDoc MP Imaging System, Bio Rad-Laboratories, Hercules, California, USA). Matrices were placed in the incubator for 48 h, with images obtained at 24 and 48 h. Matrix surface area was calculated using Axio Vision 40 × 64 V 4.9.1.0. (Carl Zeiss Microscopy GmbH, Jena, Germany).

### Cell migration

Fibroblasts (3,5 × 10^3^) suspended in a volume of 70 μl were placed in both chambers of a μ- dish insert (ibidi GmbH, Planegg, Germany) and incubated (37 °C, 5% CO_2_) for 24 h. The insert was removed using a sterile tweezer (Aesculap AG, Tuttlingen, Germany) and 1 × 1 cm hemostat slices together with 3 ml of culture medium were added. The μ- dishes were placed under a live-cell-imaging microscope (JuLI Br, NanoEnTek, Seoul, Korea) for 24 h, taking 1 picture per hour and documented the gap between the two cell fractions. Picture analysis was performed using Wound Healing Image Analysis, WimScratch (ibidi GmbH, Planegg, Germany).

### Hemostat-based pH effects

In order to identify the cellular effects resulting from post-degradation hemostat end-products, we lowered the pH of cell culture medium of controls to the pH value of ONRC (medium pH 6.6) or ORC (medium pH 7.0). We imitated pH values obtained over time, matching values for the experiments described above for each measuring point independently, utilizing a 5N HCL solution (Merck, Grafing, Germany). Differences in pH value of less than 10% for each measuring point were considered acceptable.

### Statistical analysis

The data are shown as the mean ± SD. Statistical analysis was performed using GraphPad Prism 6.0 (San Diego CA, USA). Kolmogorov–Smirnov test was used to test for the normality of data. One way ANOVA and multiple t-test with Bonferroni correction was used to compare differences between ORC, ONRC and controls (ORC, ONRC and buffered controls) in a time course for normally distributed data. Kruskal-Wallis test with Dunn’s post test and Mann–Whitney U test were applied for not normally distributed data. The level of significance was set to p < 0.05.

## Additional Information

**How to cite this article**: Wagenhäuser, M. U. *et al*. Oxidized (non)-regenerated cellulose affects fundamental cellular processes of wound healing. *Sci. Rep.* **6**, 32238; doi: 10.1038/srep32238 (2016).
